# Management of patellar tendinopathy: a systematic review and network meta-analysis of randomised studies

**DOI:** 10.1136/bmjsem-2021-001110

**Published:** 2021-11-29

**Authors:** Dimitris Challoumas, Carles Pedret, Mairiosa Biddle, Nigel Yong Boon Ng, Paul Kirwan, Blair Cooper, Patrick Nicholas, Scott Wilson, Chris Clifford, Neal L Millar

**Affiliations:** 1 Institute of Infection, Immunity and Inflammation, University of Glasgow, Glasgow, UK; 2 Sports Medicine and Imaging Department, Clinica Mapfre de Medicina del Tenis, Barcelona, Spain; 3 Department of Trauma & Orthopaedic Surgery, Queen Elizabeth University Hospital, Glasgow, UK; 4 School of Physiotherapy, Royal College of Surgeons in Ireland, Dublin, Ireland; 5 Physiotherapy Department, Connolly Hospital Blanchardstown, Blanchardstown, Ireland; 6 Department of Trauma & Orthopaedic Surgery, Ayr University Hospital, Ayr, UK; 7 Physiotherapy Department, NHS Greater Glasgow and Clyde, Glasgow, UK

**Keywords:** treatment, exercise, tendinopathy

## Abstract

**Objectives:**

We performed a systematic review and network meta-analysis (NMA) of randomised controlled trials (RCTs) to provide insights into the effectiveness of available treatment modalities in patellar tendinopathy(PT).

**Methods:**

Several databases were searched in May 2021 for RCTs assessing the effectiveness of any intervention compared with any other intervention, placebo or no treatment for pain and/or function in PT. The risk of bias and strength of evidence were assessed with the Cochrane Collaboration and GRADE (Grading of Recommendations, Assessment, Development and Evaluations)/GRADE-NMA tools.

**Results:**

A total of 37 RCTs were eligible that assessed 33 different interventions and their combinations, most represented by single studies. Based on pairwise meta-analyses of two RCTs, extracorporeal shockwave therapy (ESWT) does not appear to be superior to sham ESWT (eccentric exercise in both groups) for short-term pain (mean differences (MD) +0.1, 95% CI (−0.8 to 1), p=0.84) or function (MD −1.8, 95% CI (–8 to 4.4), p=0.57). Based on a pairwise meta-analysis of three RCTs, isometric exercise appears as effective as isotonic exercise for immediate postintervention pain relief (MD −1.03, 95% CI (−2.6 to 0.5), p=0.19). Our NMA showed that topical glyceryl trinitrate (GTN) and hyaluronic acid injection, both combined with eccentric exercise and moderate, slow resistance exercise had the highest probability of being the most effective interventions (low/very low strength of evidence).

**Conclusions:**

Promising interventions with inadequate evidence, such as topical GTN, hyaluronic acid injections and isometric and slow resistance exercise, should be further investigated through high-quality RCTs. Meanwhile, eccentric loading with or without adjuncts should remain the first-line treatment for all individuals with patellar tendinopathy.

**Summary box**

**What is already known**

- There is a multitude of treatment options available for patellar tendinopathy and no definitive guidelines exist to guide healthcare professionals.
- Eccentric exercise is usually the first-line treatment modality for patellar tendinopathy.
- Published evidence on the effectiveness of most treatment modalities remains conflicting.

**What are the new findings**

- Extracorporeal shockwave therapy appears to provide limited clinical benefits in patellar tendinopathy.
- Topical glyceryl trinitrate and hyaluronic acid injections could be useful as an adjunct if symptoms persist despite tendon loading alone for a few months.
- Heavy or moderate slow resistance and isometric exercises appear promising and should be investigated further in the future.

## Introduction

Patellar tendinopathy describes persistent pain and dysfunction of the patellar tendon related to mechanical loading.^1^ It usually occurs as a response to overuse and has a complex, multifactorial pathology. The condition is more common in athletes who participate in sports that involve repetitive loading of the tendon, such as basketball and volleyball.^2^ Across all sports, up to 22% of elite athletes report patellar tendon pain at some point during their career.^3^ Diagnosis of patellar tendinopathy is clinical, with the patient often describing activity provoked localised tendon pain and stiffness. In the early stages, an individual can often continue activity. However, the pain can progress, resulting in chronic impairment with an average duration of 32 months.^4^

Management can be divided into active and passive modalities. Active strategies mainly involve tendon-loading regimes, of which eccentric training has been the most popular, with a 50%–70% chance of improvement reported at 3–6 months follow-up.^5^ Recently, both isometric and heavy slow resistance (HSR) exercises were shown to be effective in reducing pain and improving function in patellar tendinopathy.^6^ There is a multitude of passive treatments for patellar tendinopathy, which include, but are not limited to, anti-inflammatory medications, corticosteroid and platelet-rich plasma (PRP) injections, iontophoresis, topical glyceryl trinitrate (GTN), extracorporeal shockwave therapy (ESWT), low energy laser therapy and therapeutic ultrasound (US). Surgical intervention is usually considered when non-operative interventions have been unsuccessful. Definitive guidelines on the management of patellar tendinopathy do not currently exist.

We aimed to summarise the available evidence on the management of patellar tendinopathy both with direct comparisons between interventions and by producing treatment ranks with a network meta-analysis (NMA) using direct and indirect comparisons. We expect our findings will inform the formulation of future guidelines and guide further research.

## Methods

The present systematic review has been conducted and authored according to the ‘Preferred Reporting Items for Systematic Reviews and Meta-Analyses–NMAs’ (PRISMA-NMA) guidelines.

Our population, intervention, comparator and outcomes (PICO) were defined as follows:

P: patients with patellar tendinopathy.

I: any treatment modality for patellar tendinopathy.

C: any other treatment modality, placebo or no treatment.

O: Visual Analogue Scale (VAS) or equivalent for pain (primary outcome) and Victorian Institute of Sports Assessment-Patellar (VISA-P; secondary outcome).

### Eligibility

Included studies had a randomised design of any type and compared treatment modalities for patellar tendinopathy with other treatment modalities, placebo or no treatment. Additionally, at least one of our preset outcome measures had to be included in the study. Participants had to be over 18 years of age with a clinical diagnosis of patellar tendinopathy of any duration and severity with or without radiological confirmation. Duration of the condition was not a criterion and neither were previous treatments and follow-up. Inclusion of special populations (eg, athletes) was not an exclusion criterion and was not considered in analyses, provided that their proportion in the treatment groups was comparable.

Non-randomised comparative studies, observational studies, case reports, case series, literature reviews, published conference abstracts and studies published in languages other than English were excluded.

### Search strategy

A literature search was conducted by DC and MB via Medline, EMBASE, Scopus, CENTRAL and CINAHL in May 2021, with the following Boolean operators in ‘all fields’: ‘((patellar tendinopathy) OR (jumper’s knee) OR (patellar tendinitis) OR (patellar tendinosis) OR (patellar tendonitis)) AND ((treatment) OR (management) OR (intervention) OR (exercise) OR (concentric) OR (eccentric) OR (isometric) OR (slow resistance) OR (physiotherapy) OR (electrotherapy) OR (injection) OR (steroid) OR (glyceryl) OR (gtn) OR (platelet-rich plasma) OR (prp) OR (autologous) OR (shockwave) OR (eswt) OR (polidocanol) OR (hyaluronic) OR (surgery) OR (sham))’.

Relevant review articles were screened to identify eligible articles that may have been missed during the initial search. Additionally, reference list screening and citation tracking in Google Scholar were performed for each eligible article. Clinical trial registries were also searched for ongoing or recently completed relevant studies which have not yet been published.

### Screening

From a total of 7913 articles that were initially identified, after exclusion of non-eligible articles, title and abstract screening and addition of missed studies identified subsequently, 37 studies were found to fulfil the eligibility criteria. Online supplemental figure 1 (PRISMA flowchart) illustrates the article screening process.

10.1136/bmjsem-2021-001110.supp1 Supplementary data

### Risk of bias—strength of evidence assessment

The internal validity (freedom from bias) of each included study was assessed with the ‘Cochrane Collaboration’s tool for assessing the risk of bias in randomised trials’ separately by DC and MB, and a third independent opinion (NLM) was sought where disagreements existed.^7^ Overall, studies were characterised as of ‘low’, ‘high’ or ‘unclear’ overall risk of bias with the use of the authors’ judgement based on whether they thought the possible bias identified in the subcomponents of the Cochrane tool could have influenced the true results of the study.

The strength of evidence was assessed with the GRADE tool, where the results of two or more studies were pooled in pairwise meta-analyses and with the GRADE-NMA tool for NMAs.^8 9^ Strength of evidence assessment was performed by DC and MB independently and any disagreements were resolved by discussion and involvement of a third assessor (NLM). The strength of evidence of each outcome measure within each comparison was assessed separately. Our recommendations for clinical practice were based on results of either ‘high’ or ‘moderate’ strength of evidence with both clinical and statistical significance. Clinical significance was defined as at least 1.5 points difference in pain VAS and 13 points in VISA-P between the compared interventions and these thresholds were also used as the minimal clinically relevant difference (MCRD) as part of the ‘imprecision’ risk assessment of the GRADE tool.^8^

As part of the GRADE-NMA tool, the certainty of the evidence of the direct estimate was rated first using the overall risk of bias, inconsistency (statistical heterogeneity), indirectness (clinical heterogeneity) and publication bias. Subsequently, the indirect estimate was rated using the lowest ratings of the two direct comparisons forming the most dominant first-order loops and intransitivity (differences in study characteristics of studies used in indirect comparisons). Finally, the network estimate was rated using the highest certainty of evidence between direct and indirect estimates, the incoherence (difference between direct and indirect comparisons—assessed using the ‘node splitting’ approach) and imprecision.^9^

### Data extraction—handling

The primary author (DC) extracted the key characteristics of each eligible article and inserted in tables in Microsoft Word to facilitate analysis and presentation.

For the presentation of results, outcomes were divided into short term (≤12-week follow-up), mid-term (˃12 weeks–≤12-month follow-up) and long term (˃12 months follow-up). All short-term follow-up time points were converted to weeks and mid-term follow-up time points to months for consistency and easier analysis.

Comparisons of interventions reported by two or more studies at similar follow-up time points were pooled quantitatively by pairwise meta-analyses in the absence of significant clinical heterogeneity. Raw mean differences (MD) with their accompanying 95% CI were calculated and used in the tests as the tools used across studies were the same. Finally, an NMA was conducted for both outcome measures (pain VAS and VISA-P) at each follow-up period where adequate data existed.

Where pain results were reported in different settings (eg, at rest, at night, during sports) in studies, pain during sports was preferentially used. Placebo/sham treatment was considered as no treatment in both the pairwise and NMAs.

### Statistical analysis

The Review Manager V.5 software was used for pairwise meta-analyses and accompanying forest plots and heterogeneity tests (χ^2^ and I^2^). STATA V.16.1 with Ian White’s ‘mvmeta’ extension (multivariate random effects meta-regression) was used for NMAs (frequentist approach).^10^ This uses a general model of treatment contrasts and it allows for both heterogeneity and inconsistency. A frequentist approach estimates the network model, expressing the consistency and inconsistency models as multivariate random effects meta-analysis or meta-regression.^10^

Where exact mean and SD values were not reported in the included articles, approximate values (to the nearest decimal place) were derived from the graphs. When only IQR was reported, the SD was calculated as IQR/1.35. When only median was reported, mean was assumed the same. When CIs of means were reported, SDs were calculated by dividing the length of the CI by 3.92 and then multiplying by the square root of the sample size. Where standard errors of the mean were given, these were converted to SDs by multiplying them by the square root of the sample size. In studies where only the means and the population were given, the SD was imputed using the SDs of other similar studies using the ‘prognostic method’ (calculating the average of all SDs).^11^ Pooled means were calculated by adding all the means multiplied by their sample size and then dividing this by the sum of all sample sizes. Pooled SDs were calculated with the following formula:

SD_pooled_=√ [SD_1_^2^(n_1_–1)]+[SD_2_^2^(n_2_–1)]+ … +[SD_k_^2^(n_k_–1)]/(n_1_+n_2_+ … +n_k_–k), where n=sample size and k=number of samples.

The following formula was used for the sample size calculation as part of GRADE’s assessment for imprecision:

$$N=\frac{2{\left(a+b\right)}^{2}SD^{2}}{x^{2}}$$

where

N=the sample size required in each of the groups—calculated as 34 patients for VAS and 20 for VISA-P.

x=MCRD; defined as 1.5 points for pain VAS and 13 points for VISA-P.

SD^2^=population variance (calculated using pooled SD from included treatment groups).

a=1.96 (for 5% type I error).

b=0.842 (for 80% power).

Potential publication bias was not assessed as no direct comparisons included more than 10 studies. Expecting wide-range variability in studies’ settings, a random effects model was employed in all meta-analyses.

## Results

### Study characteristics

Online supplemental table 1 shows the characteristics of the included studies.^12–48^ A total of 37 randomised controlled trials (RCTs) were found to be eligible, including 1332 patients (1407 tendons) with patellar tendinopathy (mean age 29.2 years). The majority of patients had chronic tendinopathy (>3 months) and the mean duration of symptoms, where stated, was 25.7 months (range 1–120 months). The included studies assessed 33 interventions that were applied alone or in combination with other interventions. Follow-up ranged from immediate postintervention follow-up to 53 months. The most frequent follow-up time points were 12 and 24–26 weeks. Two studies only assessed immediate postintervention outcomes. All but 5 of the included studies included pain as an outcome; 31 studies used a numerical scale of 0–10 or 0–100 (‘VAS’ or ‘Numerical Rating Scale’) and 1 a non-numerical categorical scale. VISA-P was used in 30 studies.

### Risk of bias assessment

Online supplemental table 2 shows the risk of bias assessment for each study, with the overall risk of bias as determined by the authors of the present review and justification for the high overall risk of bias decisions. Fifteen studies were considered with low and 22 with a high overall risk of bias.

### Findings—pairwise meta-analyses

Online supplemental table 3 illustrates the results of the studies, with pooling where possible (two or more studies comparing the same interventions with results at the same follow-up periods) and the strength of evidence for each outcome measure for the pooled comparisons. Justifications are provided in the table legend for downgrading the strength of evidence where the GRADE tool was used.

The findings of the pooled results from meta-analysed data are presented below. All results based on single studies can be seen in the table and are not discussed further as they are considered to be of limited evidence; those from studies with a low overall risk of bias are underlined.

### ESWT+eccentric exercise versus sham ESWT+eccentric exercise

**Figure 1:**
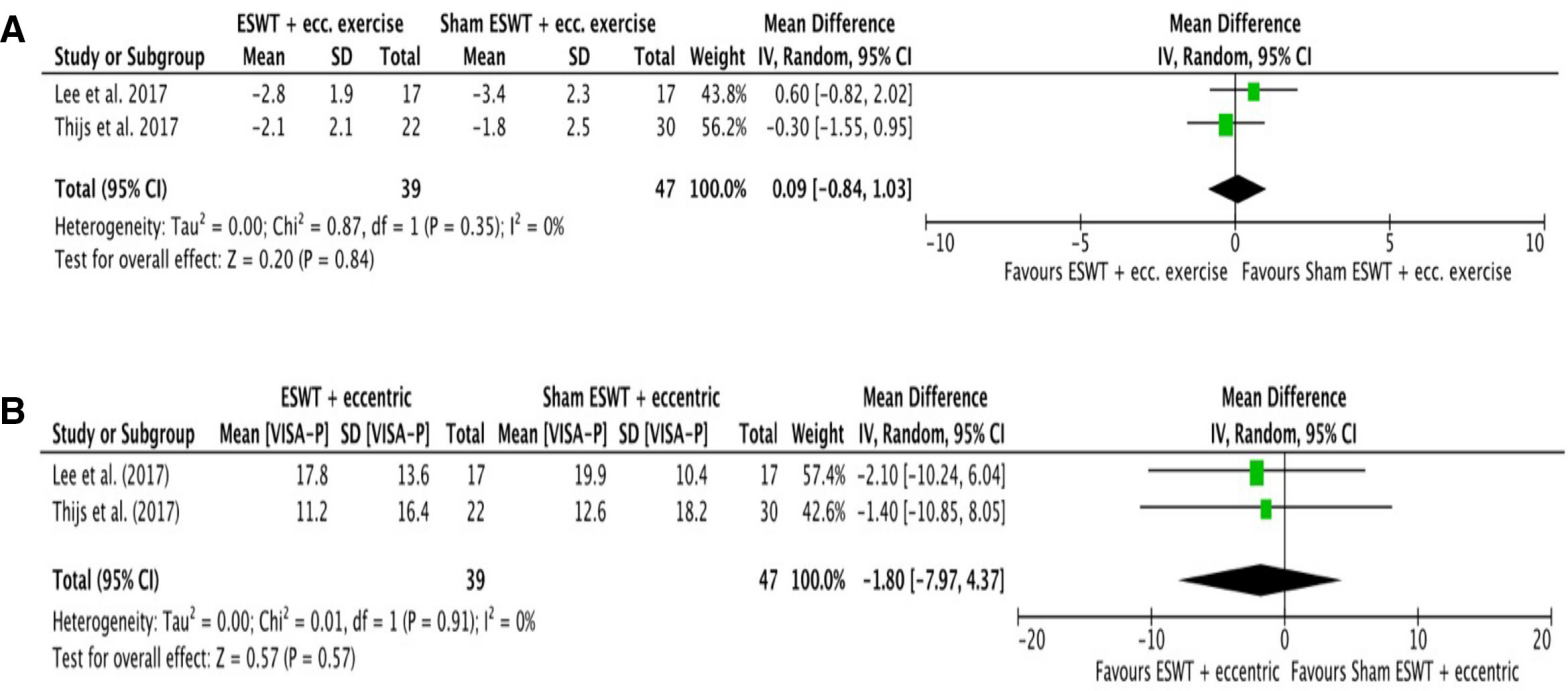
Meta-analysis results and forest plot of ‘ESWT+eccentric exercise versus sham ESWT+eccentric exercise’ comparison for (A) short-term pain and (B) VISA-P. ESWT, extracorporeal shockwave therapy; VISA-P, Victorian Institute of Sports Assessment-Patellar.

Two RCTs (one of high and one of low overall risk of bias) assessed the effectiveness of ESWT+eccentric exercise versus sham ESWT+eccentric exercise for patellar tendinopathy at short-term follow-up (12 weeks).^27 39^ The pooled result of the two studies demonstrated no statistically or clinically significant differences for pain VAS (MD +0.1 VAS points favouring sham ESWT group, 95% CI (−0.8 to 1), p=0.84, I^2^=0%) or VISA-P (MD −1.8 VISA-P points favouring sham ESWT (−8 to 4.4), p=0.57, I^2^=0%). Figure 1A, B shows the forest plot for this comparison with the numerical results of the meta-analysis for pain VAS and VISA-P, respectively.

#### Summary—strength of evidence

ESWT+eccentric exercise appears no more effective than sham ESWT+eccentric exercise for pain or functional outcomes based on moderate strength evidence.

### Isometric exercise versus isotonic exercise

**Figure 2:**
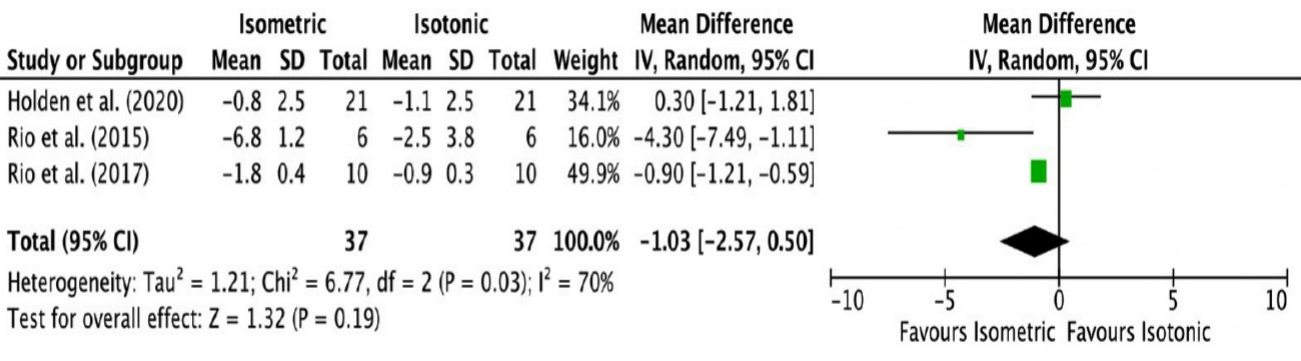
Meta-analysis results and forest plot of ‘isometric exercise versus isotonic exercise’ comparison for immediate postintervention pain.

Four studies compared isometric with isotonic exercise for patellar tendinopathy.^22 32 33 40^ One was with low and three were with a high overall risk of bias. Three of them assessed immediate postintervention outcomes.^22 32 33^ The pooled result of the three studies for immediate postintervention pain VAS showed no statistically or clinically significant differences (MD −1.03 VAS points favouring isometric group, 95% CI (−2.6 to 0.5), p=0.19, I^2^=70%). Figure 2 shows the forest plot for this comparison with the numerical results of the meta-analysis.

When the results of the fourth study with a follow-up at 4 weeks were added to the pooling to generalise the results into ‘short term’ (0–4 weeks), the meta-analysis had to be abandoned due to substantial statistical heterogeneity (I^2^=83%).

#### Summary—strength of evidence

Isometric exercise appears as effective as isotonic exercise for reducing immediate postintervention pain in patellar tendinopathy based on evidence of low strength.

### Eccentric exercise versus concentric exercise

Two RCTs with a high overall risk of bias assessed the effectiveness of eccentric versus concentric loading for patellar tendinopathy.^16 23^ This meta-analysis was abandoned due to high statistical heterogeneity (I^2^=83%); therefore, conclusions about this comparison could not be reached.

### Findings—NMA

**Figure 3:**
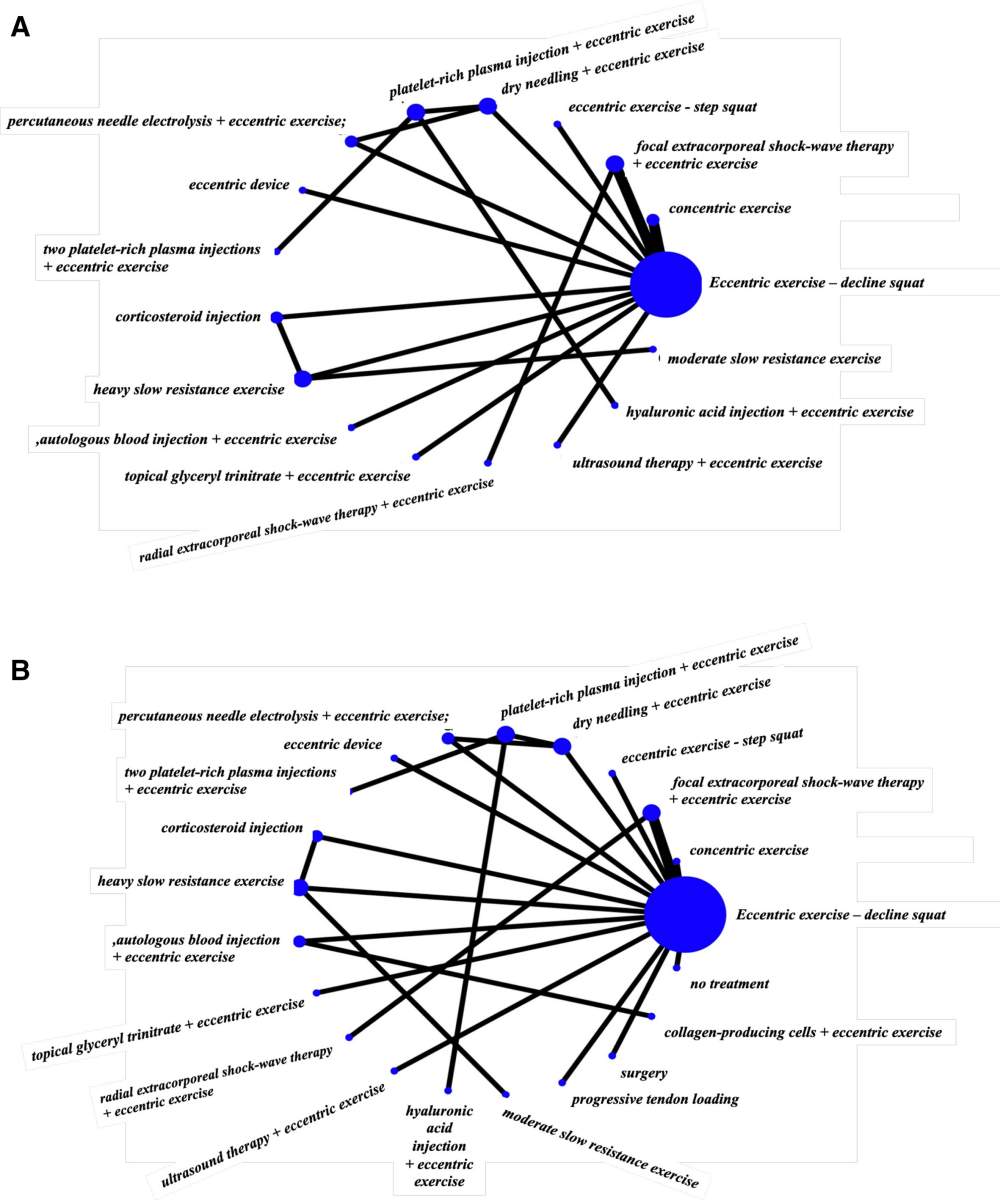
Network maps of the studies included in the network meta-analysis for (A) short-term VAS pain and (B) short-term VISA-P. The size of the circle is proportional to the number of studies that represented each intervention and the thickness of the line between interventions is proportional to the number of studies assessing that comparison. All comparisons are assessed by one study only, except for eccentric exercise–decline squat versus concentric exercise and eccentric exercise–decline squat versus focal extracorporeal shockwave therapy+eccentric exercise which are assessed by two studies. VAS, Visual Analogue Scale; VISA-P, Victorian Institute of Sports Assessment-Patellar.

**Figure 4:**
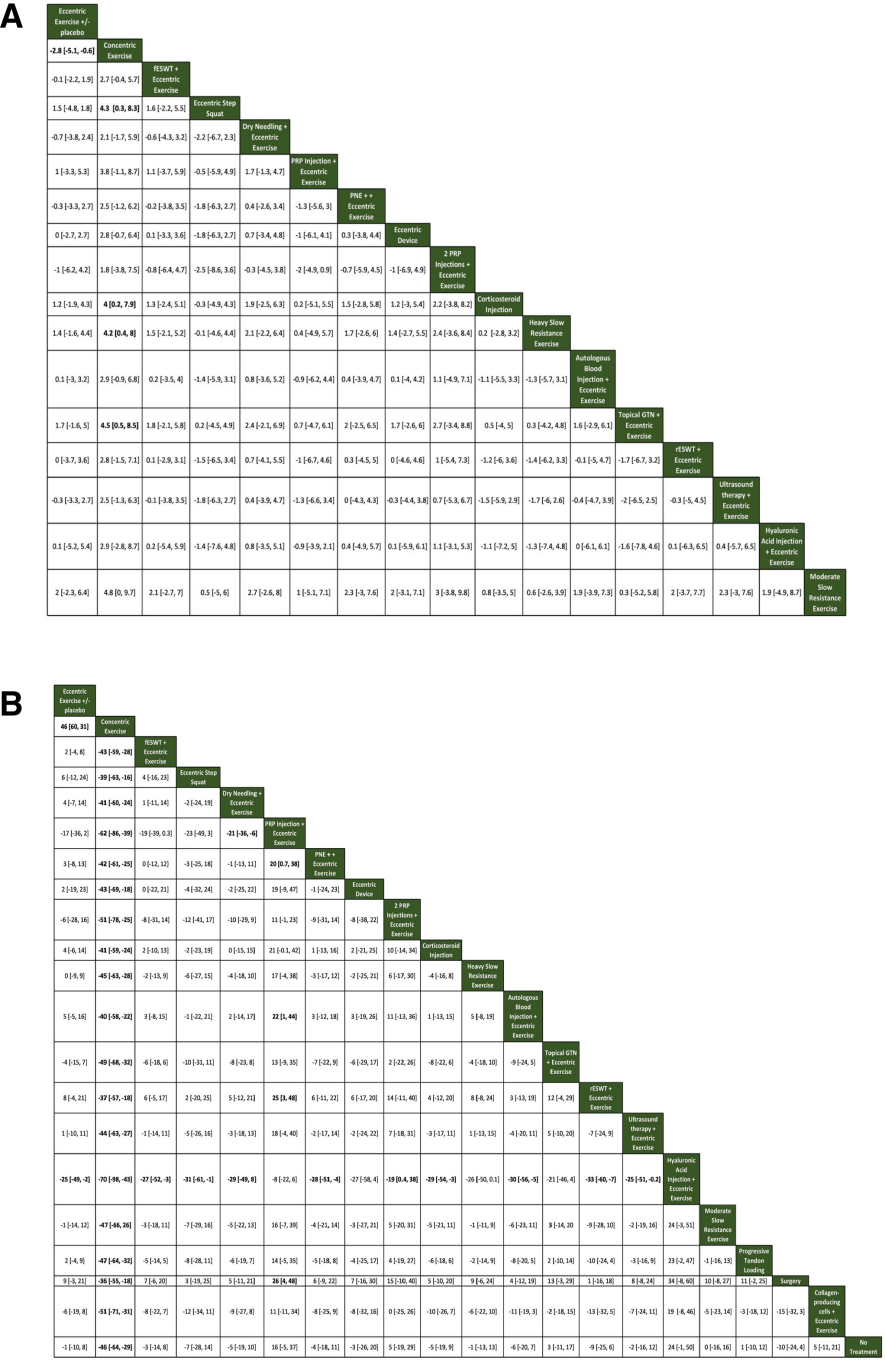
Comparative treatment class effects expressed as mean difference with 95% CI for (A) short-term pain VAS and (B) short-term VISA-P. Each cell represents the result of the comparison of the intervention of that column versus the intervention of that row. A negative value in a cell favours the column intervention in (A) and the row intervention in (B). fESWT, focal extracorporeal shock wave therapy; GTN, glyceryl trinitrate; PNE, percutaneous needle electrolysis; PRP, platelet-rich plasma; rESWT, radial extracorporeal shock wave therapy; VAS, Visual Analogue Scale; VISA-P, Victorian Institute of Sports Assessment-Patellar.

For short-term pain VAS (8–12 weeks), a total of 16 studies that were in a loop were used for the NMA, which assessed 17 different interventions (figure 3A). For short-term VISA-P (8–12 weeks), 19 studies were used for the NMA, which assessed 21 different interventions (figure 3B). Figure 4A, B illustrates the comparative treatment class effects for pain VAS and VISA-P, respectively, with MD and 95% CIs. For pain VAS (figure 4A), a negative MD in a cell favours the treatment of that column. For VISA-P (figure 4B,) a negative MD in a cell favours the treatment of that row. Values in bold represent statistically significant differences.

**Figure 5:**
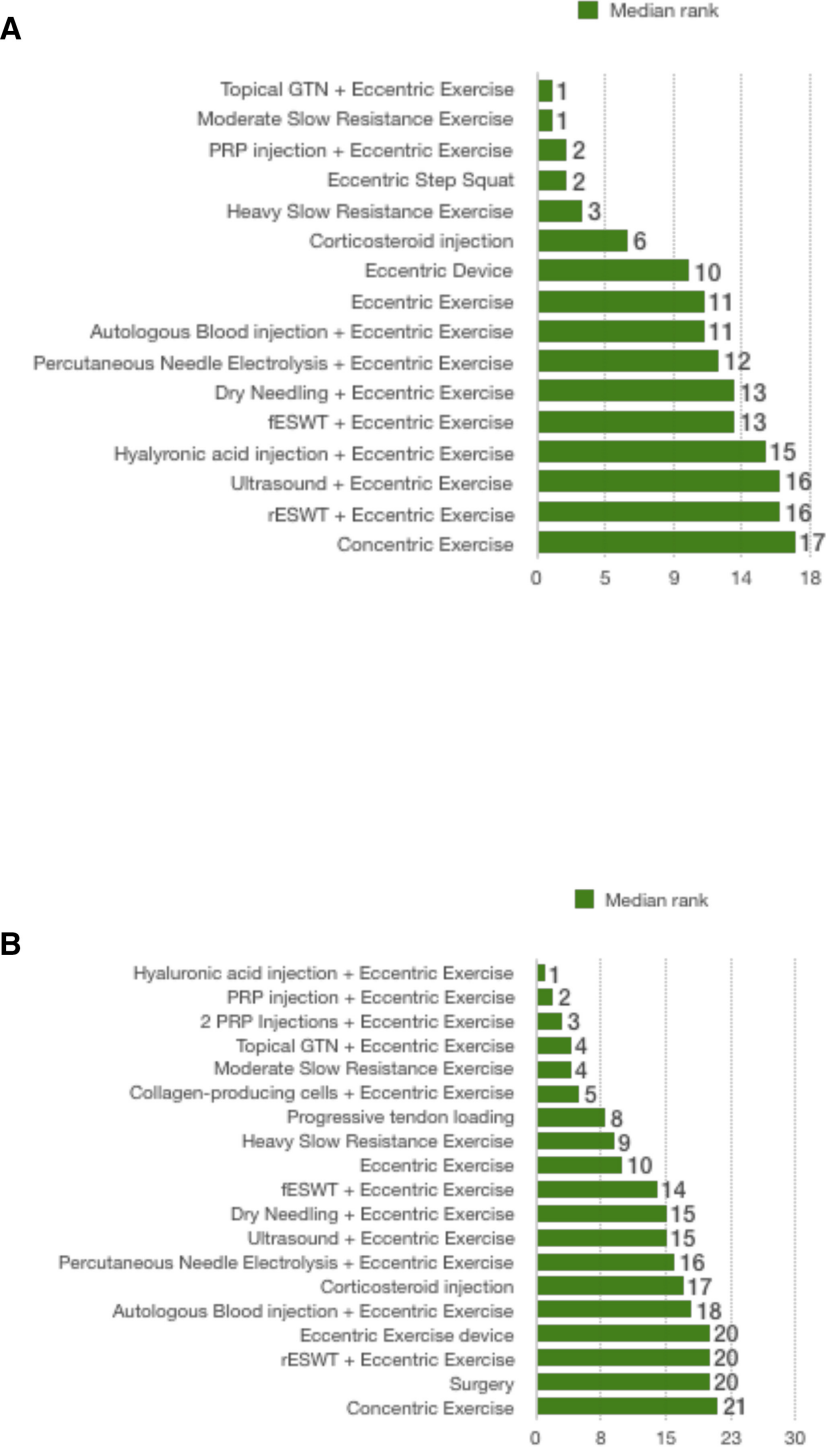
Median rank of the effectiveness of interventions included in the network meta-analysis for (A) short-term pain VAS and (B) VISA-P for chronic patellar tendinopathy. GTN, glyceryl trinitrate; fESWT, focal extracorporeal shock wave therapy; PRP, platelet-rich plasma; rESWT, radial extracorporeal shock wave therapy; VAS, Visual Analogue Scale; VISA-P, Victorian Institute of Sports Assessment-Patellar.

The interventions with the highest median rank for pain VAS were topical GTN+eccentric exercise and moderate, slow resistance exercise. In the head-to-head comparison of the two favoured topical GTN+eccentric exercises. However, the difference was not significant (0.3 VAS points (−5.2 to 5.8)) (figure 4A). The intervention with the lowest median rank was concentric exercise (figure 5A).

For VISA-P, the intervention with the highest median rank was hyaluronic acid injection+eccentric exercise and that with the lowest median rank was concentric exercise (figure 5B).

Only two comparisons included evidence from both direct and indirect estimates (eccentric exercise versus concentric exercise and ESWT+eccentric exercise versus sham ESWT+eccentric exercise). The results of the network estimates for all comparisons for both pain VAS and VISA-P were of low or very low strength of evidence due to the high overall risk of bias, imprecision and intransitivity; the only results of moderate strength of evidence were the direct estimates of the comparison between ESWT+eccentric exercise and sham ESWT+eccentric exercise presented above in the pairwise meta-analyses.

Mid-term and long-term data were inadequate for NMAs.

## Discussion

In the largest systematic review and meta-analysis of RCTs to date, the most striking finding was the absence of adequate high-quality evidence to convincingly demonstrate superior outcomes associated with specific treatment modalities. Based on evidence of moderate strength from the pairwise meta-analyses, ESWT+eccentric exercise is no more effective than sham ESWT+eccentric exercise at 12 weeks for pain or VISA-P and based on evidence of low strength isometric and isotonic exercises appear equally effective for immediate postintervention pain relief. The NMA found that treatment rankings favoured combined treatment with topical GTN and eccentric exercise, moderate, slow resistance exercise for pain and combined treatment with hyaluronic and eccentric exercise for VISA-P. Concentric exercise monotherapy had the highest probability of being the least effective treatment for both pain VAS and VISA-P. The NMA results should be interpreted with caution as they are based on studies with a high overall risk of bias; this is reflected by the low/very low strength of evidence that accompanies these results.

The effectiveness of ESWT for the treatment of patellar tendinopathy appears to be limited. Our two included RCTs comparing combined treatment with eccentric exercise and either ESWT or sham ESWT showed no short-term benefits of the former compared with the latter when short-term results were pooled (5.27). One of these studies (low overall risk of bias) reporting mid-term results did not demonstrate any superiority of ESWT versus sham ESWT for either pain or VISA-P.^5^ A further study with a low overall risk of bias compared ESWT with sham ESWT without exercise agreed with the above findings, showing no benefits of the former in the short term or mid-term for pain or VISA-P.^48^ In contrast, a single session of ESWT had favourable outcomes for VISA compared with ‘conservative treatments’ that included analgesia, patellar strap, eccentric exercise, physiotherapy (massage, phonophoresis, cold packs) and activity modification.^44^ However, this study had a high overall risk of bias, with results only having been reported ‘before and after treatment’ and not at prespecified time points.^44^ In a systematic review, Mani-Babu *et al* showed limited benefits of ESWT when compared with conservative interventions on patellar tendinopathy.^49^ Similarly, the review by Korakakis *et al* concluded that moderate strength evidence suggests that ESWT is no better than placebo ESWT.^50^ Finally, no benefits of ESWT were found compared with sham treatment for patellar tendinopathy in the short term or mid-term/long term in the review by Mendonca *et al*.^51^ Based on our findings and those of previous systematic reviews, we recommend that ESWT should not be used to treat patellar tendinopathy.

Isometric exercise has been recommended to have immediate post-treatment benefits for pain relief compared with isotonic exercise for patellar tendinopathy.^32^ Our pooled results combined the findings of three studies and found no statistically or clinically significant benefits of isometric versus isotonic exercise for postintervention pain relief. This is supported by a recent systematic review that assessed the effectiveness of isometric exercise on all tendinopathies.^52^ Additionally, based on limited evidence (single study) with a low overall risk of bias included in the present review, HSR exercise appears promising. It was found to be at least as effective as eccentric exercise in the short term and mid-term.^20^ The systematic review of studies of all evidence levels by Lim and Wong concluded that isometric exercise appears to be more effective for athletes with patellar tendinopathy during competitive seasons for short-term pain relief, while eccentric and HSR exercises are more suitable for long-term pain reduction.^6^

The effectiveness of topical GTN, as shown in our NMA, is in agreement with our previous systematic review, which found that topical GTN was more effective than placebo for the management of tendinopathies.^53^ At a short-term follow-up, topical GTN was more effective than control of pain (poor strength of evidence). Benefits were also evident for mid-term satisfaction, chances of being asymptomatic, strength and range of movement (moderate strength of evidence). Based on the results of both reviews, we recommend that topical GTN should be considered an adjunct when tendon loading alone has not been effective for 12 weeks for patellar tendinopathy. Patients should be alerted of its side effects, including skin reactions and especially headaches, which can be severe.^53^

In the first systematic review of RCTs on patellar tendinopathy, Larsson *et al* concluded that eccentric exercise appears to be the most effective treatment.^54^ US therapy was found to be ineffective.^54^ In their systematic review that was not limited to RCTs, Everhart *et al* concluded that patellar tendinopathy should initially be treated with eccentric exercise, ESWT or PRP and surgery or ESWT can be considered if no significant improvement has been observed in 6 months.^55^ In a similar systematic review that included studies of all levels of evidence, Andriolo *et al* found that multiple PRP injections may offer the most substantial long-term benefits and their use should be considered.^56^ Finally, Di Matteo *et al* and Liddle and Rodriguez-Merchan reported a lack of high-quality literature on the effectiveness of PRP on patellar tendinopathy, which precluded definitive conclusions.^57 58^ With the inclusion of observational studies, the level of evidence of these four last systematic reviews is not as high as our systematic review and others that included RCTs only.^55–58^

In the only other NMA on the management of patellar tendinopathy, Chen *et al* reported similar results to this current review from their pairwise meta-analyses, demonstrating the limited effectiveness of ESWT for pain and VISA-P.^59^ Their NMA results showed PRP and dry needling having the highest probabilities for being the most effective interventions. However, their NMA methodology has significant flaws, including studies in the network not being in a loop (ie, not all studies had common comparators), which is essential for an NMA, and the inclusion of results from different, non-specified follow-up time points. In addition, their review included only 11 studies, while ours included 37.

The value of eccentric exercise monotherapy in the management of patellar tendinopathy should not be overlooked. With the constant emergence of new therapies for tendinopathy, recent research has primarily focused on exploring the effectiveness of combined treatment with exercise and another intervention. However, we must not forget what has been consistently demonstrated to be an effective therapy for patellar tendinopathy: eccentric loading monotherapy.^14 16 20 23 54^ Several studies have historically shown the benefits of eccentric loading on patellar tendinopathy. Given its effectiveness, good tolerability and safety, it should continue to be used as first-line treatment for patellar tendinopathy.^14 16 20 23 54^

Our study is not without limitations. Despite including all published relevant RCTs to date, thorough risk of bias and strength of evidence assessments and pooling of results at prespecified follow-up time points, the lack of evidence precluded reaching conclusions with adequate certainty for most comparisons. Placebo interventions were considered and treated the same as no treatment when in reality a placebo could have independent effects on tendinopathy. However, throughout the review, we took into account exercise regimes administered to participants alongside the main interventions tested and pooled studies accordingly. Finally, although most studies included athletic individuals, the nature, level and frequency of sports were not homogeneous; this heterogeneity was recognised in our strength of evidence assessment as it may have influenced the effects of treatment modalities.

## Conclusions

Based on our findings and those of previously published systematic reviews, we recommend against the use of ESWT or concentric exercise monotherapy to manage patellar tendinopathy. Eccentric loading should continue to be used as first-line treatment and isometric loading, which may be as effective for immediate pain relief (low strength of evidence), may be preferable in select cases. However, its mid-term and long-term effects remain unknown. Heavy or moderate slow resistance exercise also appears effective and could be used instead of or in combination with eccentric exercise, although its effectiveness is only based on limited evidence. Topical GTN and/or hyaluronic acid injections could be considered adjuncts at 12 weeks if loading regimes alone have been ineffective. Finally, eccentric loading should continue to be assessed as monotherapy in future research.
