# Supplementary material for: Management of patellar tendinopathy: a systematic review and network meta-analysis of randomised studies
Source: BMJ Open Sport Exerc Med. 2021 Nov 29;7(4):e001110. doi: 10.1136/bmjsem-2021-001110 (PMC8634001; doi:10.1136/bmjsem-2021-001110)

Supplementary material

Study	Population (mean age)	Mean DOS (range)	Interventions	Duration of treatment	Follow up	Pain score	Function score
**Abat et al. (2016)**	N=64 athletes (31y)	29.2m	1)Electro-physiotherapy + eccentric exercise n=32<br>2)US-guided galvanic electrolysis + eccentric exercise n=32	8 weeks	0, 8w	-	VISA-P (0-100)
**Agergaard et al. (2021)**	N=44 athletes (30.6y)	7.1m (3-12m)	1)Moderate, slow resistance exercise n=23<br>2)Heavy slow resistance exercise n=21	Exercise for 12 weeks	0, 12w, 52w	NRS (0-10) – during sports	VISA-P (0-100)
**Bahr et al. (2006)**	N=35 athletes (40 tendons) (31y)	33m (6-100m)	1)Eccentric exercise (decline squat) n=20 tendons<br>2)Surgery n=20 tendons	Exercise for 12 weeks min	0, 12w, 26w, 52w	VAS (0-10) – during functional tests	VISA-P (0-100)
**Breda et al. (2021)**	N=76 athletes (24y)	>1y (median)	1) Progressive tendon-loading exercise n=38<br>2)Eccentric exercise n=38	Exercise for 24 weeks	0, 12w, 24w	-	VISA-P (0-100)

**Cannell et al (2001)**	N=19 athletes (26y)	3.6m (1.6-6.1m)	1)Eccentric exercise (drop squat) n=10<br>2)Concentric exercise n=9	Exercise daily for 12 weeks	0, 6w, 12w	VAS (0-10) - unspecified	-
**Clarke et al. (2011)**	N=60 tendons (46 patients) (36y) – unspecified population	11.1m	1)Injection of collagen-producing cells derived from dermal fibroblasts and suspended in autologous plasma + eccentric exercise n=33<br>2)Injection of autologous plasma + eccentric exercise n=27	Single-injection	0, 6w, 3m, 6m	-	VISA-P (0-100)
**De Vries et al. (2016)**	N=97 athletes (27y)	18m (?m-?m)	1)Patellar strap n=21<br>2)Sports taping n=18<br>3)Placebo taping n=16<br>4)No treatment n=14	1 week	0, 1w	VAS (0-100) – during and after sports	-
**Dragoo et al. (2014)**	N=23 (35y) – unspecified population	Not stated	1)Dry needling + eccentric exercise n=13<br>2)PRP + eccentric exercise n=10	A single treatment with dry needling and PRP<br>Eccentric programme for the duration of the study	0, 3w, 6w, 9w, 12w, ≥26w	VAS (0-10) - unspecified	VISA-P (0-100)
**Frohm et al. (2007)**	N=20 athletes (27y)	Not stated (>3m)	1)Eccentric device (Bromsman) n=11	Supervised eccentric exercise twice weekly for 12 weeks	Weekly. Isokinetic tests 0, 12w	VAS (0-10) - unspecified	VISA-P (0-100)

			2)Eccentric exercise (decline squat) n=9				
**Hoksrud et al. (2006)**	N=33 athletes (42 tendons) (24.8y)	37m	1)Polidocanol injections n=17<br>2)Placebo injections n=16<br>(Groups crossed over at 12w)	Single-injection<br>Further injection at 4w or 8w if persistent pain and neovascularization	0, 4m, 8m, 12m	-	VISA-P (0-100)
**Holden et al. (2020)**	N=21 athletes (26.5y)	24m (10-84m)	1) Isometric exercise n=21<br>2) Dynamic (isotonic) exercise n=21<br>The same group performed both interventions (cross-over)	A single session of each intervention	0, post-intervention	NRS (0-10) – during functional tests	-
**Jonsson et al (2005)**	N=19 tendons (15 athletes) (24.9y)	17.4m (8-72m)	1)Eccentric exercise (decline squat) n=10 tendons<br>2)Concentric exercise n=9 tendons	Exercise daily for 12 weeks	0, 12w	VAS (0-100) – during sports	VISA-P (0-100)
**Kaux et al. (2016)**	N=20 (30.3y) – unspecified population	17m	1)Single PRP injection + eccentric exercise n=10<br>2) Two PRP injections + eccentric exercise n=10	A single injection or two injections within a week	0, 6w, 3m, 12m	VAS (0-10) - unspecified	VISA-P (0-100)
**Kaux et al. (2019)**	N=33 athletes (29.4y)	>3m	1)PRP injection + eccentric exercise n=18	A single injection of PRP, 2 injections of HA 7 days apart	0, 6w, 3m	VAS (0-10) - unspecified	VISA-P (0-100)

			2)HA injection + eccentric exercise n=15				
**Kongsgaard et al. (2009)**	N=37 athletes (32.4y)	18.3m (>3-36m)	1)Corticosteroid injection n=12<br>2)Eccentric exercise n=12<br>3)Heavy slow resistance exercise n=13	2 injections over 4 weeks<br>Exercise for 12 weeks	0, 12w, 24w	VAS (0-100) – during sports	VISA-P (0-100)
**Lee et al. (2017)**	N=34 athletes (22.6y)	33.6m	1)f-ESWT + eccentric exercise n=17<br>2)Sham ESWT + eccentric exercise n=17	6 sessions of ESWT over 6 weeks<br>Eccentric exercise for 12 weeks	0, 12w	VAS (0-10) – during activity	VISA-P (0-100)
**Lopez-Royo et al. (2021)**	N=50 athletes (32.5y)	>3m	1) Dry needling + eccentric exercise n=16<br>2) Percutaneous needle electrolysis + eccentric exercise n=17<br>3) Eccentric exercise n=17	4 needling sessions over 8 weeks	0, 10w, 22w	VAS (0-10) – over last 24 hours	VISA-P (0-100)
**Pietrosimone et al. (2020)**	N=28 athletes (only N=13 used as remaining 15 asymptomatic) (19.6y)	>4m	1) Isometric exercise n=13<br>2)Sham transcutaneous electric nerve stimulator n=13	Single session	0, post-intervention	VAS (0-10) – during functional tests	-

			Groups crossed-over				
**Resteghini et al. (2016)**	N=22 athletes (38.9y)	18m (5-36m)	1)Autologous Blood Injection + eccentric exercise n=11<br>2)Placebo injection + eccentric exercise n=11	Single injection	0, 1m, 3m, 12m	VAS (0-10) - unspecified	VISA-P (0-100)
**Rigby et al. (2015)**	N=31 (24.6y) – unspecified population	>1m <2y	1)Wired iontophoresis n=11<br>2)Wireless iontophoresis n=10<br>3)Sham iontophoresis n=10	2 weeks	0, 1w, 2w	NRS (0-100) – during functional tests	-
**Rio et al. (2017)**	N=29 athletes (23y)	35.8m (1-120m)	1)Isometric exercise n=13<br>2)Isotonic exercise n=16	4 weeks	0, daily pre- and post-exercise, 4w	NRS (0-10) – during functional tests	VISA-P (0-100)
**Rio et al. (2015)**	N=6 athletes (27y)	Not stated	1)Isometric exercise n=6<br>2)Isotonic exercise n=6<br>The same group performed both interventions (cross-over)	A single session of each intervention	0, post-intervention	NRS (0-10) – during functional tests	-
**Rodas et al. (2021)**	N=20 (34y) – unspecified general population	23.6m (>4m)	1) Bone marrow mesenchymal stem cells + exercise n=10	Group 1 received a sham saline injection at day 0 and bone marrow mesenchymal	0, 6m	VAS (0-10) – during functional tests	VISA-P (0-100)

			2) LP-PRP + exercise n=10<br>The exercises included isotonic, followed by concentric and then eccentric loading	stem cells injection 23 days later<br>Group 2 received LP-PRP injections at days 0 and 23			
**Scott et al. (2019)**	N=61 athletes (32y)	2.1y (>6m)	1)LR-PRP n=20<br>2)LP-PRP n=21<br>3)Saline n=20	A single injection followed by 6 weeks of supervised exercise	0, 6w, 12w, 6m, 12m	NRS (0-10) – during activity	VISA-P (0-100)
**Stasinopoulos & Stasinopoulos (2004)**	N=30 athletes (28y)	Not stated	1)Eccentric exercise + stretching n=10<br>2)Pulsed ultrasound n=10<br>3)Transverse friction	4 weeks	0, 4w, 8w, 16w	Non-numerical scale	-
**Steunebrink et al. (2013)**	N=33 athletes (32.9y)	48w	1)Topical GTN + eccentric exercise n=16<br>2)Topical Placebo + eccentric exercise n=17	One patch daily for 12 weeks<br>Eccentric exercise for 12 weeks	0, 6w, 12w, 24w	VAS (0-10) – during sports	VISA-P (0-100)
**Taunton et al. (2003)**	N=20 athletes (?y)	Not stated	1)ESWT n=10<br>2)Placebo ESWT n=10	3 to 5 sessions over 7 weeks	0, 5w, 12w	-	VISA-P (0-100)
**Thijs et al. (2017)**	N=52 athletes (27.3y)	23m (3-120m)	1)f-ESWT + eccentric exercise n=22<br>2)Sham ESWT + eccentric exercise n=30	Exercise (decline squat) for 12 weeks<br>ESWT 3 sessions over 2 weeks	0, 6w, 12w, 24w	NRS (0-10) – during functional tests	VISA-P (0-100)

			Eccentric exercise (decline squat) twice daily for 12 weeks				
**Van Ark et al. (2016)**	N=29 athletes (23y)	35.8m (1-120m)	1)Isometric exercise n=13<br>2)Isotonic exercise n=16	4 weeks	0, 4w	NRS (0-10) – during functional tests	VISA-P (0-100)
**Van der Worp et al. (2014)**	N=43 athletes (31.1y)	35.5m (>3m)	1) f-ESWT + eccentric exercise n=21<br>2) r-ESWT + eccentric exercise n=22	3 sessions over 2 weeks	0, 7w, 14w	VAS (0-10) – during activities, sports and decline squat	VISA-P (0-100)
**Vetrano et al. (2013)**	N=46 athletes (26.9y)	18.2m (>3m)	1)LR-PRP n=23<br>2)f-ESWT n=23<br>Both groups received a home exercise programme	PRP 2 injections over 2 weeks<br>ESWT 3 sessions over 1 week	0, 2m, 6m, 12m	VAS (0-10) – during functional tests	VISA-P (0-100)
**Visnes et al (2005)**	N=29 athletes (26.6y)	73.6m	1)Eccentric exercise (decline squat) n=13<br>2)Standard training n=16	Exercise twice daily for 12 weeks	0, 1-12w, 18w, 40w	VAS (0-10) – during exercise	VISA-P (0-100)
**Wang et al. (2007)**	N=50 athletes (54 tendons) (29.8y)	13.8m (6-64m)	1)ESWT n=27<br>2)"Conservative treatments" (NSAIDs, exercise, strap, physiotherapy) n=23	A single session of ESWT<br>Duration of "conservative treatments" not stated	0, 1m, 3m, 6m, 12m, then once a year up to 53m	VAS (0-10) – on palpation and walking up and down stairs	VISA (0-100)
**Warden et al. (2008)**	N=37 (27y) – unspecified population	3.8y (>6m)	1)US + eccentric exercise n=17	US and exercise for 12 weeks	0, 12w	VAS (0-10) – during activity	VISA-P (0-100)

			2)Sham US + eccentric exercise n=20				
**Willberg et al. (2011)**	N=52 tendons (45 athletes) (26y)	22m (6-60m)	1)Sclerosing polidocanol injections n=26<br>2)Arthroscopic surgery n=26	Polidocanol maximum 3 injections 6 weeks apart<br>A single treatment with surgery	0, 2w, 6-8w, 6m, 12m	VAS (0-10) – during sports and at rest	-
**Young et al. (2005)**	N=17 athletes (27.3y)	Not stated	1)Eccentric exercise (decline squat) n=9<br>2)Eccentric exercise (step squat) n=8	12 weeks	0, 12w, 12m	VAS (0-10) – during activity	VISA-P (0-100)
**Zwerver et al. (2011)**	N=62 athletes (25y)	7.7m	1)f-ESWT n=31<br>2) Sham ESWT n=31	ESWT 3 sessions over 2 weeks	0, 1w, 12w, 22w	VAS (0-10) - during activities, sports and functional tests	VISA-P (0-100)

Suppl. Table 1. Characteristics of the included randomised controlled trials (participants, interventions, comparators and outcome measures). *ESWT, extracorporeal shockwave therapy; f-ESWT, focal ESWT; GTN, glyceryl trinitrate; m, months;HA, hyaluronic acid; LP-PRP, leucocyte-poor PRP; LR-PRP, leucocyte-rich PRP; NRS, numerical rating scale; NSAIDs, non-steroidal anti-inflammatory drugs; PRP, platelet-rich plasma; r-ESWT, radial ESWT; US, ultrasound; VAS, visual analogue scale; VISA-P, Victorian Institute of Sports Assessment – Patellar; w, weeks; y, years.*

Study (year)	Internal Validity (Cochrane's Collaboration Tool for Assessing Risk of Bias)							Overall risk	Justification
	Selection bias		*Performance bias*	*Detection bias*	*Attrition bias*	*Reporting bias*	*Other*		
	Random sequence generation	*Allocation concealment*	*Blinding of patients and staff*	*Blinding of outcome measures*	*Completeness of outcome data*	*Selective reporting*			
**Abas et al. (2016)**	Low	?	High	?	Low	High	High (no power calculation)	**High**	**Single-blinded, inappropriate reporting of results, concealment process and blinding of assessment not described**
**Agergaard et al. (2021)**	Low	Low	High	Low	Low	Low	Low	**Low**	-
**Bahr et al. (2006)**	Low	Low	High	High	Low	Low	Low	**High**	**Non-blinded**
**Breda et al. (2021)**	Low	Low	High	Low	Low	Low	Low	**Low**	-
**Cannell et al (2001)**	Low	Low	High	Low	Low	Low	High (no power calculation, small population)	**High**	**Single-blinded, small population**
**Clarke et al. (2011)**	Low	?	Low	Low	Low	Low	Low	**Low**	-
**De Vries et al. (2016)**	High	?	High	?	High	Low	Low	**High**	**Inappropriate randomisation. Single-blinded, significant loss to follow up (29%)**
**Dragoo et al. (2014)**	Low	Low	Low	Low	Low	Low	High (baseline age difference)	**Low**	-
**Frohm et al (2007)**	?	?	High	?	Low	Low	High (no power calculation, small population)	**High**	**Single-blinded, small population, randomisation/concealment process and blinding of assessment not described**
**Hoksrud et al. (2006)**	Low	Low	Low	Low	Low	Low	Low	**Low**	**(Only "treatment period 1" of study included in systematic review)**
**Holden et al. (2020)**	Low	Low	Low	High	Low	Low	Low	**Low**	-

**Jonsson et al (2005)**	?	?	High	?	High	Low	High (population not enough for power)	**High**	**Single-blinded, small population, randomisation/concealment process and blinding of assessment not described, significant loss to follow up (21%)**
**Kaux et al. (2016)**	?	?	?	?	?	Low	High (no power calculation, small population, baseline difference in pain)	**High**	**Small population, baseline differences between groups (may reflect inappropriate randomisation), no details available for selection, performance, detection and attrition bias**
**Kaux et al. (2019)**	?	?	High	High	?	Low	High (baseline difference in pain)	**High**	**Non-blinded, baseline differences between groups (may reflect inappropriate randomisation), randomisation/concealment process not described**
**Kongsgaard et al. (2009)**	Low	?	High	Low	Low	Low	Low	**Low**	**-**
**Lopez-Royo et al. (2021)**	Low	Low	Low	Low	Low	Low	Low	**Low**	**-**
**Lee et al. (2017)**	?	?	High	?	Low	Low	Low	**High**	**Single-blinded, randomisation/concealment process and blinding of assessment not described**
**Pietrosimone et al. (2020)**	Low	Low	High	Low	Low	Low	Low	**Low**	**-**
**Resteghini et al. (2016)**	Low	Low	Low	Low	?	Low	Low	**Low**	**-**
**Rigby et al. (2015)**	Low	Low	?	High	?	Low	High (baseline difference in pain, small population, no power calculation, acute and chronic tendinopathy patients)	**High**	**Single-blinded, baseline difference between groups (may reflect inappropriate randomisation), no details about completeness of outcome data, no power calculation, mixture of acute and chronic tendinopathy patients**

**Rio et al. (2017)**	Low	Low	High	High	High	Low	High (no exclusion criteria)	**High**	**Non-blinded, significant loss to follow up (38%), no exclusion criteria**
**Rio et al. (2015)**	Low	Low	High	?	Low	Low	High (acute and chronic tendinopathy patients)	**High**	**Single-blinded, no description of blinding of assessment, mixture of acute and chronic tendinopathy patients**
**Rodas et al. (2021)**	Low	?	High	Low	Low	Low	High (small population)	**High**	**Small population, concealment process not described**
**Scott et al. (2019)**	High	Low	Low	High	Low	High	Low	**High**	**Single-blinded, inappropriate randomisation, inadequate reporting of results (no p values)**
**Stasinopoulos & Stasinopoulos (2004)**	Low	?	High	Low	Low	High	High (no power calculation, small population, no baseline pain data)	**High**	**Single-blinded, small population, no baseline pain data, non-clinically relevant outcome measures (categorical pain scale)**
**Steunebrink et al. (2013)**	Low	Low	?	?	Low	Low	High (baseline difference in pain)	**High**	**Baseline difference between groups (may reflect inappropriate randomisation), blinding processes not described**
**Taunton et al. (2003)**	?	Low	High	Low	Low	Low	High (no power calculation, only mean values reported for results)	**High**	**Patients not blinded any more at 12 week follow up, randomisation process not described, small sample with no power calculation, inadequate reporting of results)**
**Thijs et al. (2017)**	Low	Low	Low	Low	Low	Low	Low	**Low**	-
**Van Ark et al. (2016)**	Low	Low	High	High	High	Low	High (acute and chronic tendinopathy patients)	**High**	**Non-blinded, significant loss to follow up (38%), no exclusion criteria**
**Van der Worp et al. (2014)**	Low	Low	Low	Low	Low	Low	High (population not enough for power)	**Low**	-
**Vetrano et al. (2013)**	Low	?	High	Low	Low	Low	? (no power calculation but large population)	**Low**	-

**Visnes et al (2005)**	High	?	High	?	Low	Low	High (no power calculation, small population)	**High**	**Inappropriate randomisation/concealment, single-blinded, blinding of assessment not described, small population**
**Wang et al. (2007)**	High	?	High	?	Low	High	Low	**High**	**Single-blinded, inappropriate randomisation, inappropriate reporting of results, concealment process and blinding of assessment not described**
**Warden et al. (2008)**	Low	Low	Low	Low	High	Low	High (no power calculation, small sample)	**Low**	-
**Willberg et al. (2011)**	Low	Low	High	High	Low	Low	Low	**High**	**Non-blinded**
**Young et al. (2005)**	Low	Low	High	Low	Low	Low	High (no power calculation, small population)	**High**	**Single-blinded, small population**
**Zwerver et al. (2011)**	Low	Low	Low	Low	Low	Low	High (population not enough for power but large)	**Low**	-

Suppl. Table 2. Risk of bias assessment for the included studies with justification where studies deemed as high overall risk of bias.

Comparison	Study	Pain (VAS 0-10)			MD VAS (95% CI)			Function (VISA-P 0-100)			MD VISA-P (95% CI)		
	**Follow up**	**ST**	**MT**	**LT**	**ST**	**MT**	**LT**	**ST**	**MT**	**LT**	**ST**	**MT**	**LT**
**Eccentric (1) vs Concentric (2) exercise**	Cannell et al (2001)	↔	-	-	-1.2 (-2.4, 0)	-	-	-	-	-	-	-	-
	Jonsson et al (2005)	↓	-	-	-4.4 (-5.6, -3.2)	-	-	↑	-	-	+44.6 (35, 54.2)	-	-
**Overall Eccentric (1) vs Concentric (2) exercise (Certainty of Evidence)**		↔ **(low)**	-	-	**-2.8 (-5.9, 0.3)^ (low)**	-	-	-	-	-	-	-	-
**f-ESWT + Eccentric exercise (1) vs sham ESWT + Eccentric exercise (2)**	Lee et al. (2017)	↔	-	-	+0.6 (-0.8, 2.1)	-	-	↔	-	-	-2.1 (-7.8, 3.6)	-	-
	Thijs et al. (2017)	↔	↔	-	-0.3 (-1.6, 1)	+0.3 (-0.6, 1.2)	-	↔	↔	-	-1.5 (-8.3, 4.3)	-2.9 (-9.1, 3.3)	-
**Overall f-ESWT + Eccentric exercise (1) vs sham ESWT + Eccentric exercise (2) (Certainty of Evidence)**		↔ **(moderate)^^**	**-**	**-**	**+0.1 (-0.8, 1) (moderate)^^**	**-**	**-**	↔ **(moderate)^^**	**-**	**-**	**-1.8 (-8, 4.4) (moderate)^^**	**-**	**-**
**Isometric (1) vs Isotonic (2) exercise – immediate post-intervention outcomes**	Holden et al. (2020)	↔*	-	-	+0.3 (1.3, -0.7)	-	-	-	-	-	-	-	-
	Rio et al. (2015)	↓*	-	-	-4.3 (-7.4, -1.2)	-	-	-	-	-	-	-	-
	Rio et al. (2017)	↓*	-	-	-0.9 (-1.1, -0.7)	-	-	↔	-	-	N/A	-	-
**Overall Isometric (1) vs Isotonic (2) exercise – immediate post-intervention outcomes (Certainty of Evidence)**		↔ **(low)^^^**	**-**	**-**	**-1** (-2.6, 0.5) **(low)^^^**	**-**	**-**	**-**	**-**	**-**	**-**	**-**	**-**
**Isometric exercise (1) vs sham TENS (2) –**	Pietrosimone et al. (2020)	↔	-	-	-0.1 [-1.1, 0.9]	-	-	-	-	-	-	-	-

**immediate post-intervention outcomes**													
**Isometric (1) vs Isotonic (2) exercise – 4-week outcomes**	Van Ark et al. (2016)	↔	-	-	-0.5 (-2.6, 1.6)	-	-	↔	-	-	-1 (-11.1, 9.1)	-	-
**Moderate, slow resistance (1) vs heavy, slow resistance (2) exercise**	Agergaard et al. (2021)	↔	-	↔	-0.6 (-1.3, 0.1)	-	-0.5 (-1, 0)	↔	-	↔	+0.9 (-12.8, 14.6)	-	+1.8 (-11.8, 15.4)
**Eccentric exercise decline squat (1) vs standard/step squat (2)**	Young et al. (2005)	↑	-	↔	+1.4 (-1.3, 3.5)	-	+0.2 (-0.7, 1.1)	↔	-	↑	+7 (-1.6, 15.6)	-	+9 (0.4, 17.6)
**Progressive tendon-loading exercise (1) vs eccentric exercise (2)**	Breda et al. (2021)	-	-	-	-	-	-	↔	↑	-	+1.4 (-5.5, 8.3)	+8.7 (1.2, 16.2)	-
**Eccentric exercise (1) vs Surgery (2)**	Bahr et al. (2006)	-	-	↔	-	-	+0.8 (-0.1, 1.7)	↔	↔	-	+9 (0.9, 17.1)	0 (-8.6, 8.6)	-
**LR-PRP injection + exercise (1) vs Saline injection + exercise (2**	Scott et al. (2019)	↔	↔	↔*	+1.1 (-0.7, 2.9)	+0.8 (-0.9, 2.5)	+2.6 (0.8, 4.4)	↔	↔*	↔*	-6 (-20.9, 8.9)	-17 (-32.3, -1.7)	-22 (-38.7, -5.3)
**LP-PRP injection + exercise (1) vs Saline injection + exercise (2)**	Scott et al. (2019)	↔	↔*	↔	-1.1 (-3, 0.8)	-1.9 (-3.7, -1.1)	-0.5 (-2.3, 1.3)	↔	↔	↔	+2 (-13.8, 17.8)	+1 (-14.3, 16.3)	-5 (-20.8, 10.8)
**LR-PRP injection + exercise (1) vs LP-PRP injection + exercise (2)**	Scott et al. (2019)	↔*	↔*	↔*	+2.2 (0.4, 4)	+2.7 (1, 4.4)	+3.1 (1.2, 5)	↔	↔*	↔*	-8 (-24.7, 8.7)	-17 (-33.7, -0.3)	-17 (-35.4, 1.4)

**BM-MSC + exercise vs LP-PRP injection + exercise (2)**	Rodas et al. (2021)	-	↔	-	-	+1.3 (-0.7, 3.3)	-	-	↔	-	-	-2.2 (-23.9, 19.5)	-
**Patellar strap (1) vs Sports taping (2)**	De Vries et al. (2016)	↔	-	-	+0.2 (-0.8, 1.2)	-	-	-	-	-	-	-	-
**Patellar strap (1) vs no treatment (2)**	De Vries et al. (2016)	↔	-	-	-0.3 (-1.3, 0.7)	-	-	-	-	-	-	-	-
**Sports taping (1) vs no treatment (2)**	De Vries et al. (2016)	↔	-	-	-0.5 (-1.5, 0.5)	-	-	-	-	-	-	-	-
**Electrophysiotherapy + eccentric exercise (1) vs USGT + eccentric exercise (2)**	Abat et al. (2016)	-	-	-	-	-	-	↓	-	-	-12.5 (-21.4, -3.6)	-	-
**Dry Needling + Eccentric exercise (1) vs PRP + Eccentric exercise (2)**	Dragoo et al. (2014)	↔	↔	-	+1.5 (0, 3)	+1.1 (-0.1, 2.3)	-	↓	↔	-	-20.2 (-31.3, -10.1)	+3.3 (-8.2, 14.4)	-
**Dry Needling + Eccentric exercise (1) vs PNE + Eccentric exercise (2)**	Lopez-Royo et al. (2021)	↔	↔	-	+0.4 (-0.6, 1.4)	-0.4 (-1.3, 0.5)	-	↔	↔	-	-0.1 (-6.7, 6.5)	-3.4 (-12.5, 5.7)	-
**Dry Needling + Eccentric exercise (1) vs Eccentric exercise (2)**	Lopez-Royo et al. (2021)	↔	↔	-	+0.7 (-0.4, 1.8)	-0.5 (-1.5, 0.5)	-	↔	↔	-	-4.3 (-12, 3.4)	-3.2 (-11.4, 5)	-
**PNE + Eccentric exercise (1) vs Eccentric exercise (2)**	Lopez-Royo et al. (2021)	↔	↔	-	+0.3 (-0.7, 1.3)	-0.1 (-1.2, 1)	-	↔	↔	-	-3.6 (-11.3, 4.1)	+0.8 (-7.3, 8.9)	-
**PRP injection + eccentric exercise (1)**	Kaux et al. (2019)	↔	-	-	-0.9 (-1.9, 0.1)	-	-	↔	-	-	-8 (-17.8, 1.8)	-	-

**vs HA injections + eccentric exercise (2)**													
**Polidocanol injections (1) vs placebo injections (2)**	Hoksrud et al. (2006)	-	-	-	-	-	-	-	↑	-	-	+10 (0.1, 19.9)	-
**Injection of collagen-producing cells + eccentric exercise vs Injection of autologous plasma + eccentric exercise**	Clarke et al. (2011)	-	-	-	-	-	-	↑	↑	-	+11 (5.1, 16.9)	+8.1 (2.4, 13.7)	-
**Eccentric exercise (1) vs Eccentric device (overload) (2)**	Frohm et al (2007)	↔	-	-	0 (-0.6, 0.6)	-	-	↔	-	-	-2 (-15.6, 11.4)	-	-
**Single PRP injection + Eccentric exercise (1) vs two PRP injections + Eccentric exercise (2)**	Kaux et al. (2016)	↔*	-	↔*	-2 (1.2, 2.8)	-	-3.4 (-4.1, -2.7)	↔	-	↔	+11 (2.3, 19.7)	-	-1 (-9.1, 7.1)
**Corticosteroid injection (1) vs Eccentric exercise (2)**	Kongsgaard et al. (2009)	-**	↔	-	-1.2 (0, 2.4)	0 (-1.2, 1.2)	-	-**	↓	-	-4 (-10.9, 2.9)	-23 (-32.4, -13.8)	-
**Corticosteroid injection (1) vs Heavy Slow Resistance exercise (2)**	Kongsgaard et al. (2009)	-**	↑	-	+0.2	+2.1	-	-**	↓	-	-4 (-13.1, 5.1)	-30 (-38.9, -21.1)	-
**Heavy Slow Resistance (1) vs Eccentric (2) exercise**	Kongsgaard et al. (2009)	-**	↔	-	-1.4 (-2.5, -0.3)	-1.1 (-2.2, 0)	-	-**	↔	-	0 (-6.5, 6.5)	-7 (-14.6, 0.4)	-

**Autologous blood + eccentric (1) vs Saline + eccentric (2)**	Resteghini et al. (2016)	↔	-	↔	+0.3 (-1.9, 2.5)	-	+0.6 (-1.6, 2.8)	↔	-	↔	+4.2 (-10, 18.4)	-	-0.6 (-14.8, 13.6)
**Wired (1) vs Wireless (2) iontophoresis**	Rigby et al. (2015)	↔	-	-	-0.5 (-2.1, 1.1)	-	-	-	-	-	-	-	-
**Wired (1) vs Sham (2) iontophoresis**	Rigby et al. (2015)	↔	-	-	-1.1 (-3.1, 1.1)	-	-	-	-	-	-	-	-
**Wireless (1) vs Sham (2) iontophoresis**	Rigby et al. (2015)	↔	-	-	-1.6 (-3.4, 0.2)	-	-	-	-	-	-	-	-
**Eccentric exercise + stretching (1) vs pulsed US (2)**	Stasinopoulos & Stasinopoulos (2004)	↓	-	-	-	-	-	-	-	-	-	-	-
**Eccentric exercise + stretching (1) vs transverse friction (2)**	Stasinopoulos & Stasinopoulos (2004)	↓	-	-	-	-	-	-	-	-	-	-	-
**Pulsed US (1) vs transverse friction (2)**	Stasinopoulos & Stasinopoulos (2004)	↔	-	-	-	-	-	-	-	-	-	-	-
**Topical GTN + Eccentric exercise (1) vs Placebo + Eccentric exercise (2)**	Steunebrink et al. (2013)	-	↔	-	-1.7 (-3.1, -0.3)	-0.5 (-2, 1)	-	-	↔	-	+4.1 (-3.7, 11.9)	-0.9 (-8.9, 7.1)	-
**f-ESWT + Eccentric exercise (1) vs r-ESWT + Eccentric exercise (2)**	Van der Worp et al. (2014)	↔	-	-	+0.1 (-1, 1.2)	-	-	↔	-	-	+6.3 (-1.6, 14.2)	-	-

**LR-PRP (1) vs ESWT (2)**	Vetrano et al. (2013)	↔	↓	↓	-1 (-1.8, -0.2)	-1.8 (-2.6, -1)	-2 (-2.8, -1.2)	↔	↑	↑	+5.7 (-1.2, 12.6)	+13.8 (-7.2, 20)	+14.5 (8, 21)
**Eccentric exercise (1) vs Standard training (2)**	Visnes et al (2005)	-	-	-	-	-	-	↔	-	-	-1 (-9.2, 7.2)	-	-
**ESWT (1) vs "Conservative treatments" (2)**	Wang et al. (2007)	-	-	↓	-	-	-4.7 (-5.7, -3.7)	-	-	↑	-	-	+47.7 (39.8, 55.6)
**US + Eccentric exercise (1) vs Sham US + Eccentric Exercise (2)**	Warden et al. (2008)	↔	-	-	+0.3 (-0.8, 1.4)	-	-	↔	-	-	-1.1 (-8.7, 6.5)	-	-
**Sclerosing polidocanol injections (1) vs Arthroscopic surgery (2)**	Willberg et al. (2011)	-	-	↑	-	-	-	-	-	-	-	-	-
**f-ESWT (1) vs Sham ESWT (2)**	Zwerver et al. (2011)	↔	↔	-	-0.2 (-1.2, 0.8)	-0.4 (-1.4, 0.6)	-	↔	↔	-	+0.8 (-4.7, 6.3)	+0.8 (-4.7, 6.3)	-
	Taunton et al. (2003)	-	-	-	-	-	-	↑	-	-	+3.7 (CI n/a)	-	-

Suppl. Table 3. Results of compared interventions from included studies shown a) qualitatively based on the direction of effect ["Pain (VAS 0-10)" and "Function (VISA-P 0-100)"] and b) quantitatively ["MD VAS (95% CI)" and "MD VISA-P (95% CI)"]. Where mean differences (MD) are reported, for pain VAS the minus (-) sign favours intervention "1" and the plus (+) sign intervention "2"; for VISA-P, the plus (+) sign favours intervention "1" and the minus (-) sign intervention "2". Where the direction of effect is reported, for pain VAS, the "down" arrow (↓) favours intervention "1" at statistical significance, the "up" arrow (↑) intervention "2" and the "equal" arrow (↔) shows no statistical difference for the compared interventions; for VISA-P, the "up" arrow (↑) favours intervention "1" and the "down" (↓) arrow intervention "2". Underlined comparisons and studies are those of low risk of bias.

BM-MSC, bone marrow mesenchymal stem cells; ESWT, extracorporeal shockwave therapy; f-ESWT, focal ESWT; GTN, glyceryl trinitrate; m, months; LP-PRP, leucocyte-poor PRP; LR-PRP, leucocyte-rich PRP; NRS, numerical rating scale; NSAIDs, non-steroidal anti-inflammatory drugs; PNE, percutaneous needle electrolysis; PRP, platelet-rich plasma; r-ESWT, radial ESWT; TENS, transcutaneous electric nerve stimulation; US, ultrasound; USGT, ultrasound-guided galvanic therapy; VAS, visual analogue scale; VISA-P, Victorian Institute of Sports Assessment – Patellar; w, weeks; y, years

^Meta-analysis abandoned due to substantial heterogeneity (I^2=83%)

^^ Strength of evidence downgraded for high overall risk of bias

^^^ Strength of evidence downgraded for high overall risk of bias and indirectness of evidence

*Differences appear significant both clinically and statistically. However, the authors state that there were no statistically significant differences

**No inter-group statistical tests at 12 weeks

N/A, not available

Figure 1. PRISMA 2009 Flow Diagram

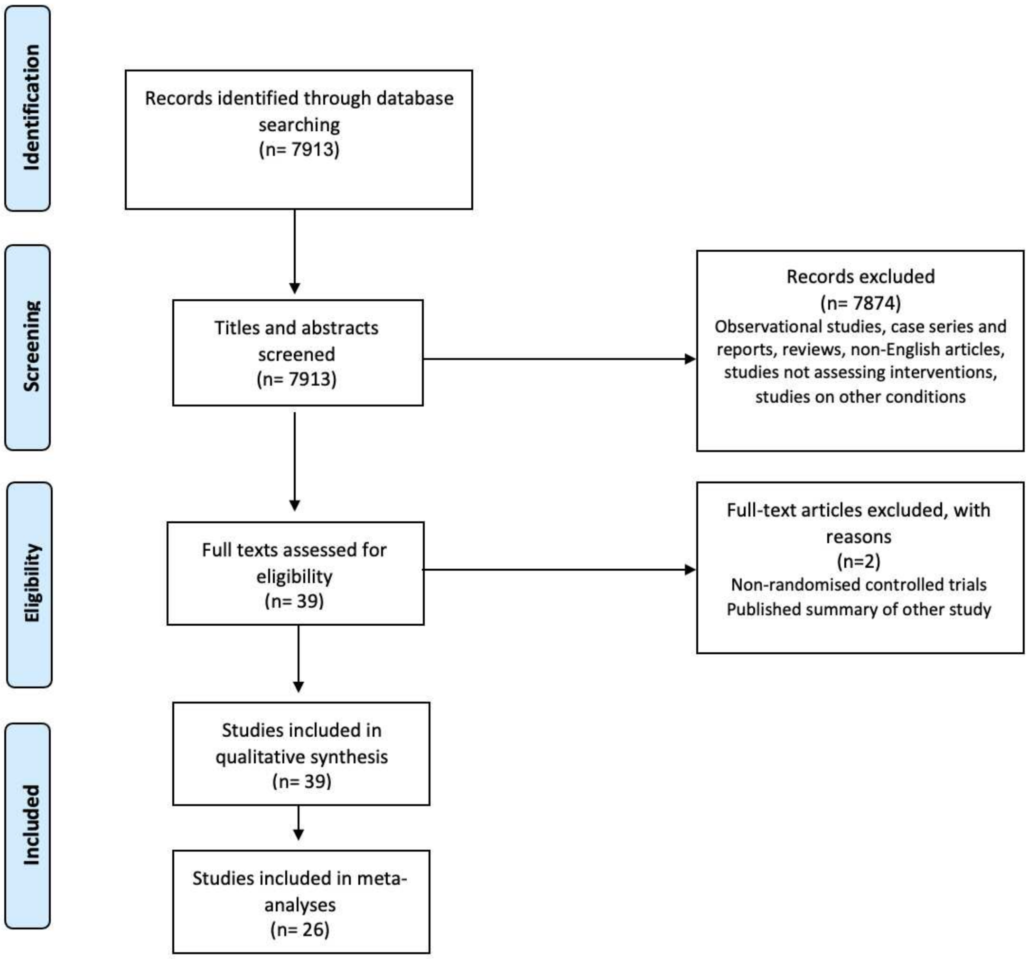

From: Moher D, Liberati A, Tetzlaff J, Altman DG, The PRISMA Group (2009). *Preferred Reporting Items for Systematic Reviews and Meta-Analyses*: The PRISMA Statement. PLoS Med 6(7): e1000097. doi:10.1371/journal.pmed1000097

Suppl. Figure 1. PRISMA flowchart showing the article selection process

Suppl. Figure 2a&b. Network forest plots for all included interventions in the network meta-analysis for short-term pain VAS (2a) and VISA-P (2b). *A, eccentric exercise – decline squat; B, concentric exercise; C, focal extracorporeal shock-wave therapy + eccentric exercise; D, eccentric exercise - step squat; E, dry needling + eccentric exercise; F, platelet-rich plasma injection + eccentric exercise; G, percutaneous needle electrolysis + eccentric exercise; H, eccentric device; I, two platelet-rich plasma injections + eccentric exercise; J, corticosteroid injection; K, heavy slow resistance exercise; L, autologous blood injection + eccentric exercise; M, topical glyceryl trinitrate + eccentric exercise; N, radial extracorporeal shock-wave therapy + eccentric exercise; O, ultrasound therapy + eccentric exercise; P, hyaluronic acid injection + eccentric exercise; Q, moderate slow resistance exercise; R, progressive tendon loading; S, surgery; T, collagen-producing cells + eccentric exercise; U, no treatment.*

2a

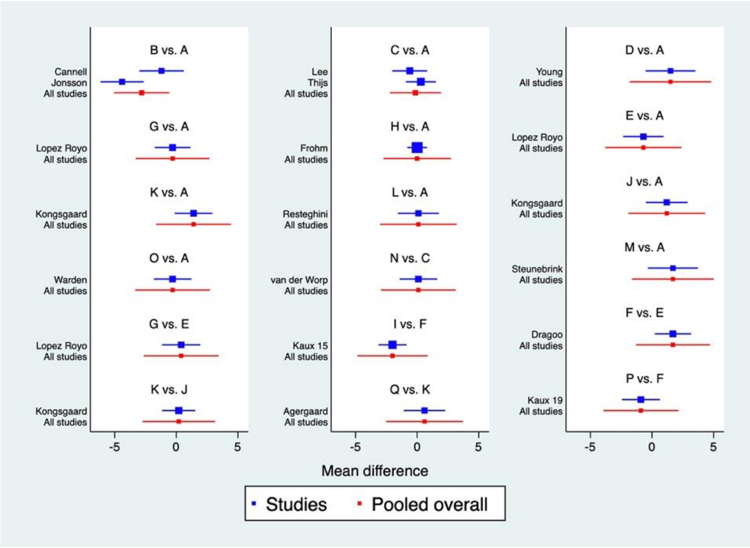

2b

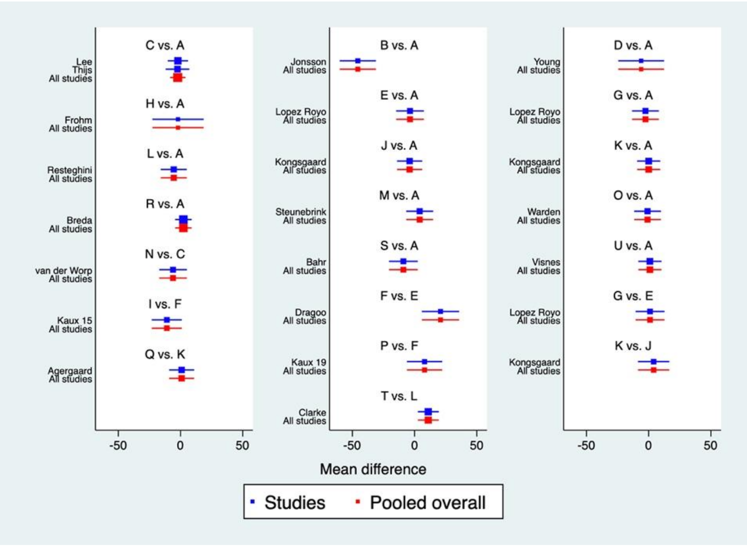

Suppl. Figure 3a&b. Network rank line showing probabilities of each intervention ranking at each position for their effectiveness for short-term pain VAS (3a) and VISA-P (3b).

A, eccentric exercise – decline squat; B, concentric exercise; C, focal extracorporeal shock-wave therapy + eccentric exercise; D, eccentric exercise - step squat; E, dry needling + eccentric exercise; F, platelet-rich plasma injection + eccentric exercise; G, percutaneous needle electrolysis + eccentric exercise; H, eccentric device; I, two platelet-rich plasma injections + eccentric exercise; J, corticosteroid injection; K, heavy slow resistance exercise; L, autologous blood injection + eccentric exercise; M, topical glyceryl trinitrate + eccentric exercise; N, radial extracorporeal shock-wave therapy + eccentric exercise; O, ultrasound therapy + eccentric exercise; P, hyaluronic acid injection + eccentric exercise; Q, moderate slow resistance exercise; R, progressive tendon loading; S, surgery; T, collagen-producing cells + eccentric exercise; U, no treatment.

3a

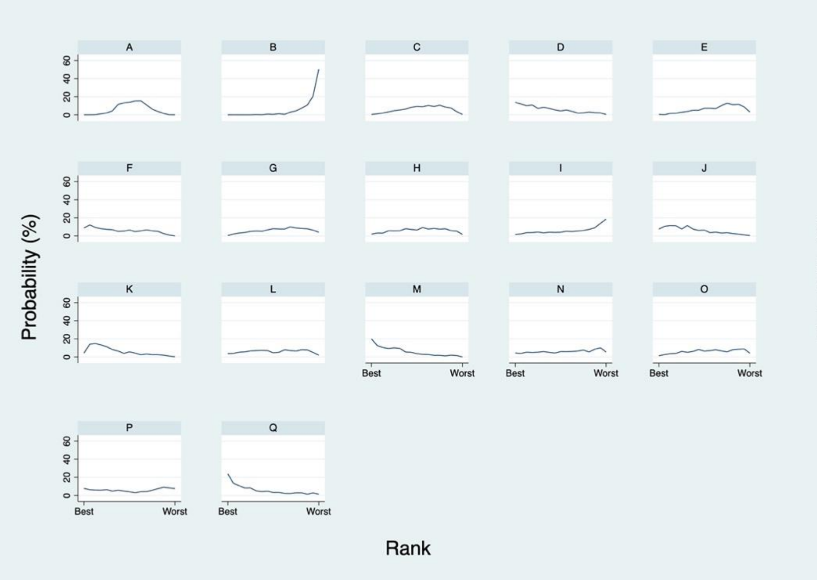

3b

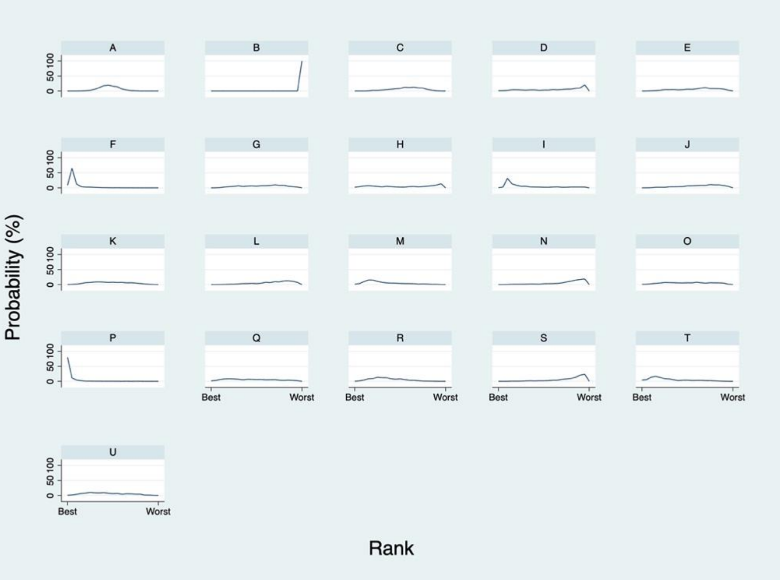

Supplement: Supplementary data [file bmjsem-2021-001110supp001.pdf]
